# Aripiprazole-Induced Priapism in a Pediatric Patient: A Case Report

**DOI:** 10.7759/cureus.92909

**Published:** 2025-09-22

**Authors:** Sebastian G Tobia Gonzalez, Gaayana Raju, Leon I Smith-Harrison

**Affiliations:** 1 Urology, Driscoll Children's Hospital, Corpus Christi, USA

**Keywords:** adverse drug reaction, antipsychotic medications, aripiprazole, atypical antipsychotic, conservative management, drug-induced priapism, extrapyramidal symptoms, ischemic priapism, pediatric urology, priapism

## Abstract

Drug-induced non-ischemic priapism is a rare but critical condition in children. Aripiprazole, an atypical antipsychotic, has been associated with priapism, mostly in adults. Prompt recognition is essential to prevent permanent sequelae.

An 8-year-old boy, with a complex past medical history including prior dystonic reactions, presented with acute dystonia and subsequent priapism following inadvertent administration of Aripiprazole. The erection persisted for approximately five hours, partially responsive to Diphenhydramine and Benztropine. Urologic evaluation confirmed ischemic priapism. Conservative management was successful, and no surgical intervention was needed. Doppler ultrasound of the penis showed normal flow without thrombosis. The patient remained clinically stable, experiencing only brief, intermittent episodes of erection, each of which resolved with conservative management.

This case underscores the potential for Aripiprazole-induced priapism in the pediatric population, despite its generally favorable safety profile. Early recognition and timely urologic consultation are essential for optimal outcomes. When identified promptly, conservative management may be effective in avoiding invasive intervention.

## Introduction

Priapism is an uncommon urological emergency in children, most often associated with sickle cell disease and other hematologic disorders [1-5]. In patients without such conditions, drug-induced priapism is a leading cause, although it remains rare. Several psychotropic agents have been implicated, including risperidone, quetiapine, lithium, atomoxetine, and oxcarbazepine.

Aripiprazole, a third-generation atypical antipsychotic, is widely prescribed in pediatric psychiatry because it is generally well tolerated and carries a lower risk of weight gain and metabolic disturbances compared to other antipsychotics [6]. Nonetheless, rare adverse effects, such as priapism, have been documented. While adult cases are reported, pediatric cases remain exceedingly scarce [7].

This report describes an unusual case of aripiprazole-induced priapism in a child with a complex medical history, highlighting the importance of vigilance, early recognition, and adherence to standard management guidelines.

## Case presentation

An 8-year-old boy with a history of attention-deficit/hyperactivity disorder, oppositional defiant disorder, dextro-transposition of the great arteries (status post arterial switch), gastrostomy dependence, and bilateral vocal cord and diaphragmatic paralysis was admitted with acute dystonic reactions and simultaneous onset of priapism following inadvertent re-administration of aripiprazole by his caregiver.

Medication history: Aripiprazole had been initiated several months earlier for behavioral regulation but was discontinued after the patient developed extrapyramidal side effects. No recent dose changes had been made, and no other medications were added or withdrawn during this period. Clonidine, previously prescribed for sleep disturbances, had been withheld and was restarted during hospitalization.

At presentation, the patient exhibited dystonic movements together with a painful, rigid penile erection. The priapism persisted for approximately five hours. Diphenhydramine provided only transient relief, and benztropine was ineffective. Lorazepam was administered for sedation.

**Figure 1:**
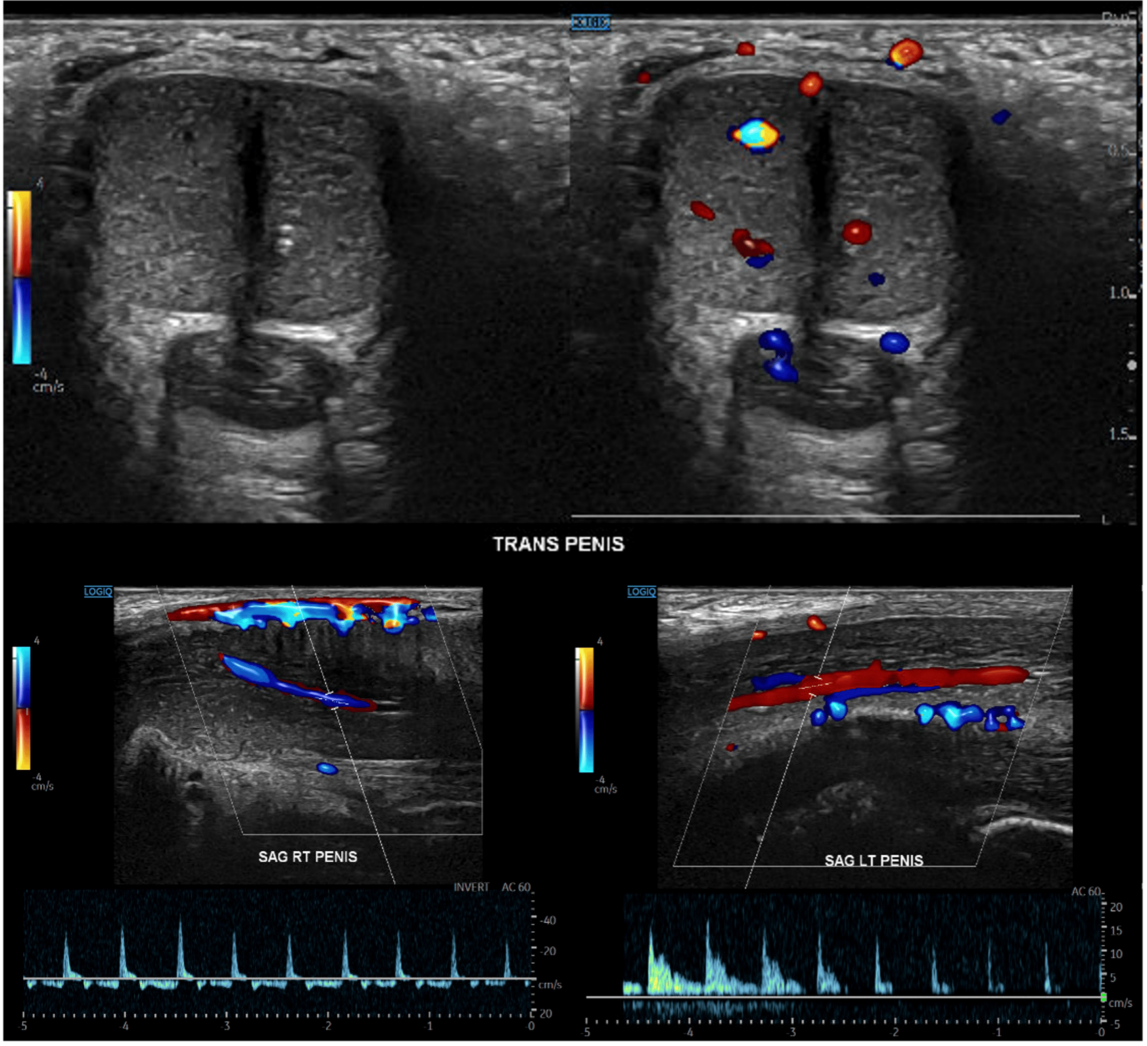
Doppler evaluation Penile Doppler ultrasonography demonstrated preserved arterial and venous blood flow without evidence of thrombotic complications or vascular compromise.

Physical examination showed a rigid, tender, non-pulsatile penis. Penile Doppler ultrasonography demonstrated preserved arterial and venous flow without thrombosis, findings that seemed inconsistent with the clinical impression (Figure 1). However, based on the painful, prolonged rigidity, the episode was diagnosed as ischemic priapism, likely a partial or stuttering variant, reconciling the Doppler findings with the clinical scenario.

Management was conservative, consisting of sedation, cold compresses, and discontinuation of aripiprazole. Pediatric urology consultation confirmed no immediate need for surgical intervention. The patient gradually improved, and clonidine was reintroduced. Following resolution of the acute event, only brief, self-limited erections (<15 minutes) occurred. Laboratory investigations (CBC, CMP, creatine kinase) were normal throughout.

## Discussion

Ischemic priapism results from impaired venous drainage and subsequent hypoxia within the corpora cavernosa [3,8,9]. In children without hematologic disorders, drug-induced priapism is a predominant cause. Aripiprazole-induced priapism is estimated to occur in approximately 1 in 10,000 adults, but pediatric cases remain rare [7,10].

The pathophysiology is incompletely understood. Proposed mechanisms include alpha-adrenergic antagonism and dopaminergic modulation, both of which may disturb vascular tone and detumescence [11-13]. In this case, the close temporal relationship between accidental re-exposure to aripiprazole and the simultaneous onset of dystonia and priapism strongly supports a causal role [14].

The discrepancy between clinical ischemia and Doppler findings emphasizes the complexity of pediatric cases. The episode was interpreted as a stuttering or partial ischemic variant, a scenario where tissue hypoxia occurs despite preserved arterial inflow.

According to the American Urological Association (AUA) and European Association of Urology (EAU) guidelines, ischemic priapism requires urgent treatment to prevent long-term erectile dysfunction [3,8,15]. First-line interventions include aspiration of corporal blood, saline irrigation, and intracavernosal phenylephrine. Surgical shunting is reserved for refractory cases. In this patient, conservative therapy was effective, but clinicians must remain prepared to escalate promptly if symptoms persist. Expert consensus reports, such as the AFUD Thought Leader Panel, also highlight the need for timely intervention to preserve erectile function [16,17,18].

Drug-induced priapism in children has been reported with multiple psychotropics, including stimulants and atomoxetine [16]. Although aripiprazole is generally considered to have a more favorable metabolic and prolactin-related safety profile compared to other antipsychotics [6,17], this case demonstrates that even agents with relatively low risk can rarely cause serious adverse effects such as priapism. Few published reports involve aripiprazole specifically in pediatrics, underscoring the rarity of this event. Moreover, many reports lack detailed dosing or follow-up data, limiting generalizability [18]. This case contributes by documenting the temporal relationship, detailed clinical course, and follow-up in a medically complex child.

Lessons learned by this case include:

(i) Thorough medication history, including previous adverse reactions and recent dose adjustments, is essential. (ii) Clinicians must maintain a high index of suspicion for priapism in children presenting with genitourinary complaints during psychotropic treatment. (iii) Early urologic consultation and close monitoring are critical. (iv) Conservative management may suffice in selected cases, but adherence to standardized protocols is mandatory when ischemia persists.

## Conclusions

This case illustrates that aripiprazole, though widely considered safe in pediatric populations, can precipitate priapism, even in patients without hematologic risk factors.

Given the single-patient nature of this report, management recommendations cannot be generalized. However, such reports enhance pharmacovigilance and increase clinician preparedness for rare but potentially serious drug reactions in children.
